# N6-methyladenosine modification and post-translational modification of epithelial–mesenchymal transition in colorectal cancer

**DOI:** 10.1007/s12672-024-01048-3

**Published:** 2024-06-04

**Authors:** Yingnan Wang, Yufan Chen, Miaomiao Zhao

**Affiliations:** Stomatology Hospital, School of Stomatology, Zhejiang University School of Medicine, Zhejiang Provincial Clinical Research Center for Oral Diseases, Key Laboratory of Oral Biomedical Research of Zhejiang Province, Cancer Center of Zhejiang University, Engineering Research Center of Oral Biomaterials and Devices of Zhejiang Province, Hangzhou, 310000 China

**Keywords:** Epithelial–mesenchymal transition (EMT), Epigenetics, Post-translational modification (PTM), N6-methyladenosine (M6A), Colorectal cancer, Tumorigenesis, Metastasis, Immunotherapy

## Abstract

Colorectal cancer is a leading cause of cancer-related mortality worldwide. Traditionally, colorectal cancer has been recognized as a disease caused by genetic mutations. However, recent studies have revealed the significant role of epigenetic alterations in the progression of colorectal cancer. Epithelial–mesenchymal transition, a critical step in cancer cell metastasis, has been found to be closely associated with the tumor microenvironment and immune factors, thereby playing a crucial role in many kinds of biological behaviors of cancers. In this review, we explored the impact of N6-methyladenosine and post-translational modifications (like methylation, acetylation, ubiquitination, SUMOylation, glycosylation, etc.) on the process of epithelial–mesenchymal transition in colorectal cancer and the epigenetic regulation for the transcription factors and pathways correlated to epithelial-mesenchymal transition. Furthermore, we emphasized that the complex regulation of epithelial-mesenchymal transition by epigenetics can provide new strategies for overcoming drug resistance and improving treatment outcomes. This review aims to provide important scientific evidence for the prevention and treatment of colorectal cancer based on epigenetic modifications.

## Introduction

Colorectal cancer (CRC) stands as a prominent contributor to cancer-related mortality on a global scale. According to estimations from the International Agency for Research on Cancer, in 2020, CRC accounted for approximately 1.8 million new cases (approximately 10% of all cancer cases), ranking third following breast and lung cancers [1, 2]. CRC is widely recognized as a disease driven by genetic alterations, involving genetic variations such as KRAS, BRAF, PIK3CA, PTEN, APC, SMAD4 and TP53 mutations. However, in recent years, extensive research focusing on epigenetic regulation has revealed that mutations only play a partial role in CRC carcinogenesis, with epigenetic changes also exerting significant influence. These findings challenge the previous understanding that mutations alone dictate the development of CRC, which indicates that epigenetic regulation plays an important role in tumorigenesis, metastasis and therapeutic application of CRC [3–8].

Despite advancements in the diagnosis and treatment of various cancers, metastasis and recurrence remain the leading causes of cancer-related deaths. To suppress cancer cell metastasis and recurrence, researchers have extensively explored the progression of epithelial-mesenchymal transition (EMT), including EMT transcription factors, signaling factors, and epigenetic regulation. In this regard, studies focusing on the epigenetic level have gained increasing attention. In-depth investigations into the mechanisms of epigenetic modifications, especially N6-methyladenosine (M6A) modification and post-translational modification (PTM) of proteins, in CRC, particularly their impact on EMT processes, will contribute to a better understanding of the pathogenesis of CRC and the development of novel therapeutic strategies, providing critical scientific foundations for the prevention and treatment of CRC worldwide.

## Role of EMT in tumor tumorigenesis and metastasis

EMT is a dynamic biological process in which epithelial cells lose cell–cell adhesion, apical-basal polarity, and their characteristic cytoskeletal structure, becoming more motile and invasive. Consequently, the acquisition of a migratory and invasive mesenchymal phenotype by epithelial cells is considered the initial and crucial step in cancer cell metastasis. Here, we explore the intricate correlation between EMT and tumorigenesis. During the EMT process, epithelial cells undergo changes, becoming more migratory and invasive. Simultaneously, EMT also influences the tumor microenvironment (TME), cancer stem cells (CSCs) and circulating tumor cells (CTCs), thereby impacting the progression of cancer. The reversibility and adaptability of the EMT process highlight its significance in both tumor tumorigenesis and therapeutic considerations.

### Initiation process of EMT

E-cadherin, a Ca^2+^-dependent cell-cell adhesion molecule, plays a significant role in epithelial tissues. Loss of E-cadherin function allows epithelial tumor cells to transition into highly migratory and invasive cells. Therefore, the loss of E-cadherin and its inhibitory or weakened effect on cell adhesion during EMT are considered critical steps. In EMT, three different types of transcription factors (TFs) are upregulated, namely SNAIL, Twist family, and ZEB. Upregulation of these factors leads to the loss of E-cadherin expression [9]. For example, SNAIL1, SNAIL2, SNAIL3, TWIST1, and TWIST2 can downregulate the expression of epithelial genes and upregulate the expression of mesenchymal genes. ZEB1 and ZEB2 can activate or repress transcription by binding to E-box regulatory sequences [10]. These TFs play a critical role in EMT-induced cancer progression, including tumor initiation, metastasis, and drug resistance associated with cancer stem cells.

Upon the blockade of E-cadherin expression, various signaling pathways such as TGF-β, Wnt/β-catenin, Hedgehog, Notch, and TNF can induce cancer cell EMT through Twist/Snail/Slug/ZEB1 [11]. Furthermore, studies have demonstrated that other signaling molecules, including Wnt proteins, growth factors, and ECM, can respond to microenvironmental stimuli and induce these programs, serving as molecular switches during the EMT process. It is evident that the initiation process of EMT involves a multitude of associated factors and pathways, with some of them extensively reported to be regulated by epigenetics. These factors and pathways will be further discussed in the subsequent sections.

### The correlation between EMT and immune factors in the tumor microenvironment

There is a close interaction between EMT and immune factors in the TME. During the progression of tumor cells towards EMT, the composition of TME undergoes changes in different stromal components. Particularly, there is a significant increase in immune cell infiltration, including monocytes and macrophages, as well as an increase in blood and lymphatic vessel density [12]. EMT-associated injuries have been shown to induce pronounced inflammatory responses, leading to the secretion of numerous soluble paracrine signals, such as TNF, IL-6, and IL-1β. In turn, these inflammatory cells can act as inducers of EMT by secreting cytokines, thus creating an appropriate tumor microenvironment for tumor metastasis. For instance, IL6/IL-6R has been demonstrated to induce integrin β6 expression in a concentration-dependent manner, activating TGF-β and promoting migration on interstitial fibronectin, thereby facilitating EMT [13, 14]. Meanwhile, macrophages can decrease the proportion of EpCAM^+^ epithelial tumor cells and early mixed EMT status, while promoting the progression of EMT towards a fully mesenchymal state [11].

Simultaneously, the interaction between EMT and immune factors in the TME plays a pivotal role in tumor resistance and immune evasion. One study indicated that high levels of EMT-related factor Brachyury not only decrease the sensitivity of cancer cells to T lymphocytes but also reduce sensitivity to NK cell-mediated lysis and cell death induced by lymphokine-activated killer cells, FAS, and TRAIL [15]. However, another study found that overexpression of EMT transcription factor SNAIL in colon cancer cells actually increased their sensitivity to NK cell-mediated lysis [16]. This suggests that the mechanisms involving immune factors in EMT and TME are highly complex and warrant further exploration. Such investigations hold significant potential in providing crucial insights for the treatment of tumors.

### The correlation between EMT and CSCs

CSCs can develop resistance to chemotherapy and radiation therapy, leading to tumor recurrence. Early study has indicated that cells derived from EMT exhibit similar differentiation potential to MSCs, which has garnered significant attention within the scientific community [17]. Following researches have demonstrated that EMT-TFs can induce the generation of CSCs and upregulate different gene products in CSCs and their differentiated progeny. This connection between EMT and CSCs is believed to have significant implications for tumor progression, as confirmed in breast cancer and prostate cancer [18, 19]. In CRC, LGR5^+^ CSCs have been shown to play a critical role in metastasis and colonization [20]. Moreover, increasing evidence supports the crucial role of EMT-TFs as activators of CSCs in tumor initiation [21]. For instance, a study reported a close association between the human BMI1 gene, EMT, and CSCs, suggesting the potential of small molecule BMI1 inhibitors as anticancer agents [22]. The connection between EMT and CSCs will propel the development of therapeutic strategies targeting CSCs, thereby underscoring the indispensable role of EMT in cancer treatment.

### The correlation between EMT and CTCs

**Fig. 1:**
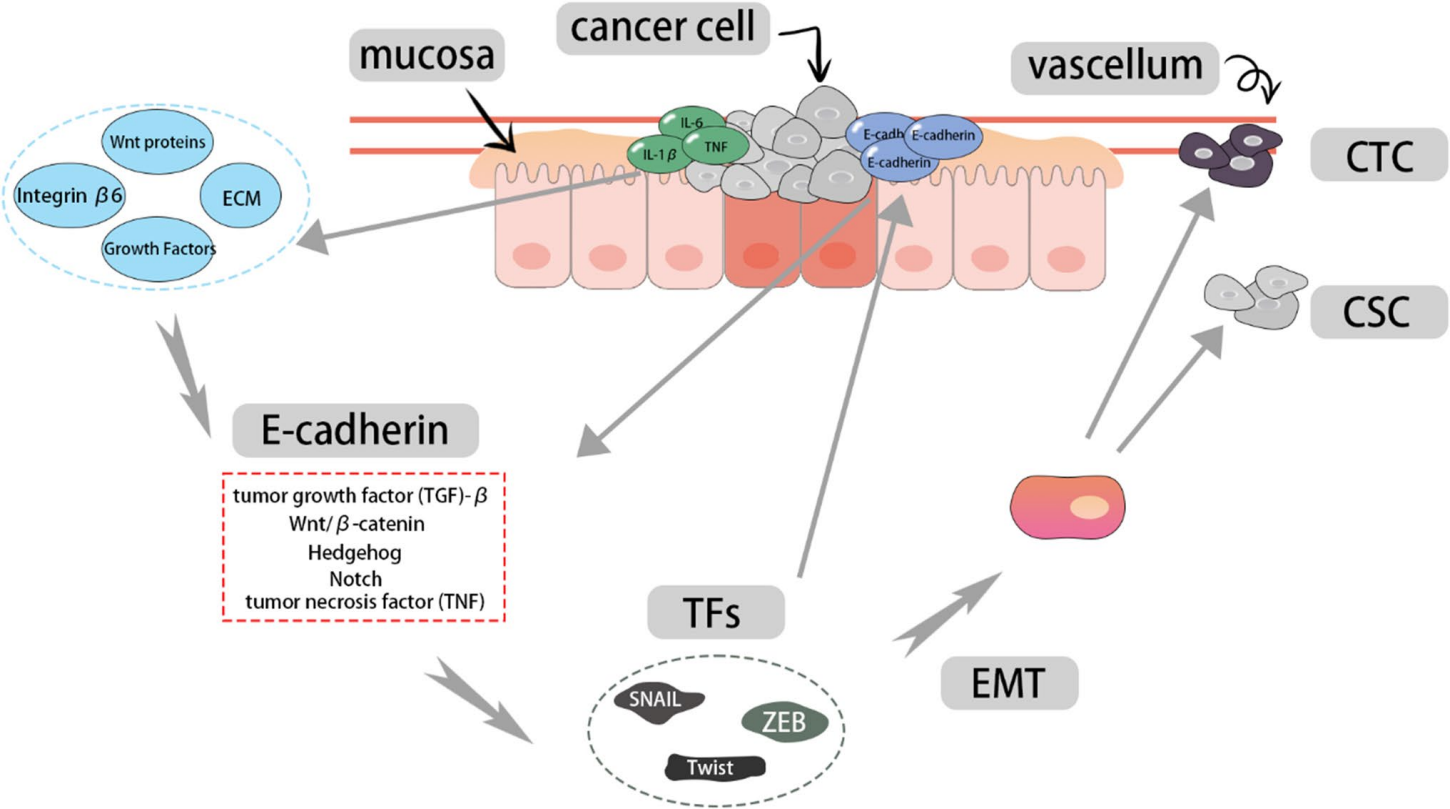
Correlation between EMT and immune factors, CSC and CTC in TME. Upregulation of TFs leads to the downregulation of E-cadherin, subsequently activating TGF-β, Wnt/β-catenin, TNF, etc., which promotes the generation of CTCs and CSCs, thereby increasing tumor drug resistance and treatment complexity. Moreover, on one hand, Wnt proteins, growth factors, and ECM components can respond to microenvironmental stimuli. On the other hand, the induction and secretion of a plethora of soluble paracrine signals, such as TNF, IL-6, and IL-1β, facilitates the progression of EMT

CTCs are cancer cells that have disseminated from the primary tumor and spread to various organs through the bloodstream or lymphatic system. The process of their dissemination and establishment is a crucial step in cancer cell metastasis [23]. Currently, CTCs are widely used in cancer assessment, including CRC [24]. As a multidimensional and nonlinear dynamic process, EMT has been demonstrated to be associated with the dissemination of CTCs. Firstly, EMT can enhance the adaptability of tumor cells to restrictive environments during the metastatic process. For instance, EMT can stimulate angiogenesis through VEGF-A [25, 26]. Secondly, experiments by Labelle et al. found that following transient contact between platelets and tumor cells, the latter adopt a phenotype characterized by increased mesenchymal and invasive properties. Concurrently, levels of E-cadherin protein decrease, indicative of a transformation and invasive behavior akin to the EMT process. Their further findings suggest that the pro-metastatic effect of platelets is largely mediated through activation of the TGF-β signaling pathway, with platelets serving as a critical source of TGF-β available to tumor cells within the vasculature. Hence, platelets may influence tumor cell behavior and induce EMT [27]. Besides, Nicola Aceto et al. proposed that the EMT features observed in CTCs not only reflect their inherent invasiveness but also indicate the influence of survival and drug resistance pathways triggered by therapeutic interventions [28]. The enhancing effect of EMT on the dissemination capability of CTCs provides a solid theoretical basis for the targeted inhibition of CTCs. Additionally, the specific biomarkers of EMT hold great potential for future clinical monitoring of CTCs (Fig. 1).

## M6A regulation of EMT in CRC

**Fig. 2:**
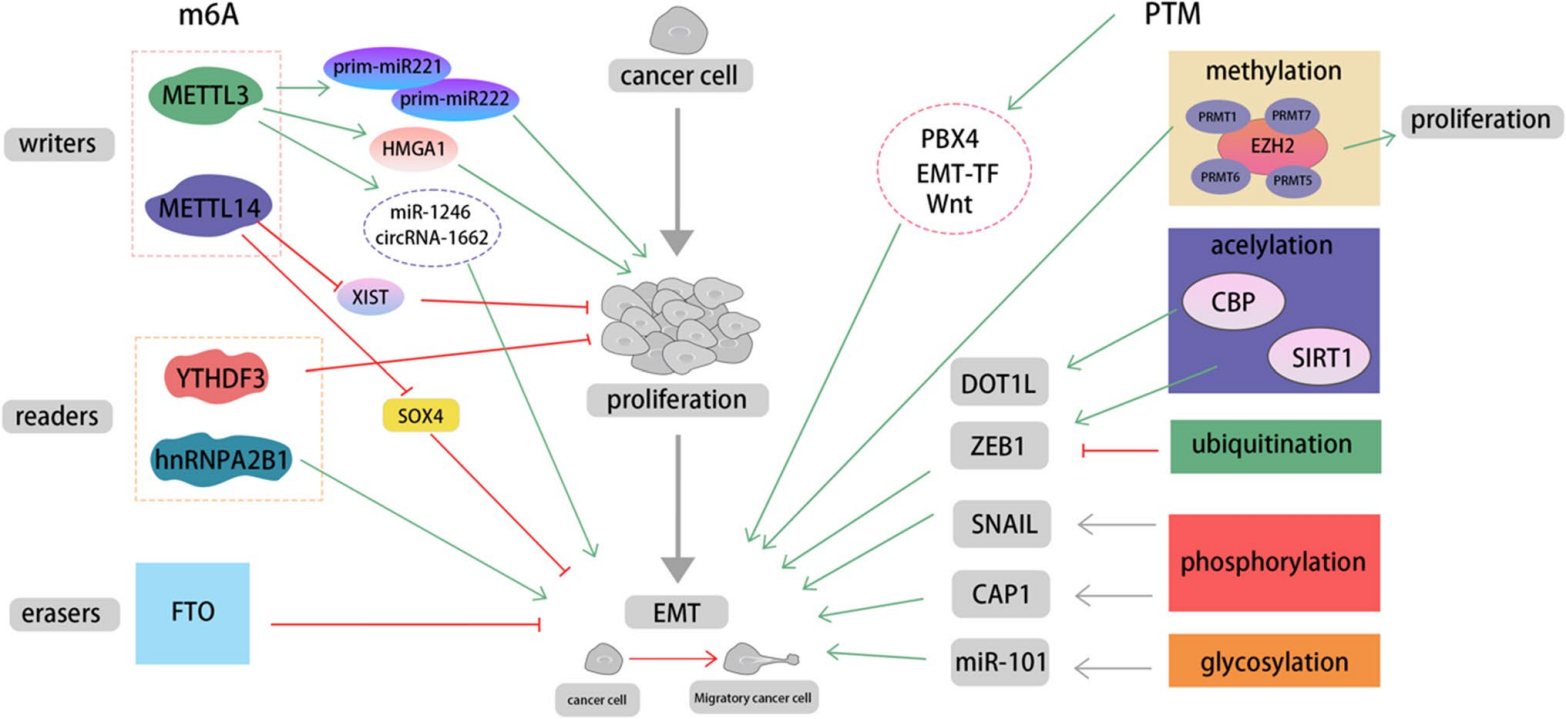
Fig.2 Regulation of tumor EMT by M6A and PTM. Within the M6A modification, the “writers”, METTL3 and METTL4, promote or inhibit cancer proliferation and metastasis by modifying their target sites; the “readers”, YTHDF3 and hnRNPA2B1, inhibit cancer cell proliferation and EMT respectively; and the “erasers”, FTO, when knocked out, promotes EMT. PTM can affect the functions of EMT-TF, Wnt, PBX4, thereby indirectly affecting EMT. In addition, in methylation, upregulation of EZH2 promotes proliferation and PRMT family promote EMT; in acetylation, CBP and SIRT1 promote the expression of DOT1L and ZEB1 to promote EMT; while ubiquitination inhibits the expression of ZEB1 and then inhibit EMT; the phosphorylation of SNAIL and CAP1 promote EMT; and the glycosylation of miR-101 can enhance the function of EZH2 protein, thereby promoting EMT

Epigenetic modifications have been widely implicated in the regulation of EMT, a key process in tumor development and metastasis. In this section, we delve into the specific role of M6A modification in the regulation of EMT. M6A modification is a prevalent form of internal RNA modification closely associated with gene and protein regulation. By binding to target mRNAs, M6A modification can induce mRNA degradation or post-transcriptional repression, thereby regulating the expression levels of target proteins. One notable study conducted by Zhan Zhao et al. demonstrated that upregulated M6A modification enhances the stability of oncogenic long non-coding RNA (lncRNA), thereby facilitating the proliferation, invasion, and migration of CRC cells [29]. The M6A modification process is regulated by three key factors: methyltransferases (“writers”), demethylases (“erasers”), and M6A-binding proteins (“readers”). These factors collectively influence the tumorigenesis and metastasis of CRC (Fig. 2).

### Methyltransferases: the writers of M6A

Methyltransferases, particularly METTL3 and METTL14, play a pivotal role in M6A modification, influencing both the development and metastasis of CRC. METTL3 is responsible for the addition of methyl groups to specific adenosine sites on RNA, while METTL14 provides structural support and aids METTL3 in recognizing RNA substrates [30]. The coordinated activity of these “writers” is essential for the completion of the M6A modification process, which holds significant implications for tumorigenesis and metastasis in various cancers.

For instance, METTL3-mediated M6A modification has been observed to stimulate the proliferation of bladder cancer cells by facilitating the maturation of prim-miR221 and prim-miR222 [31]. Furthermore, in CRC, METTL3-mediated M6A modification enhances the expression of HMGA1 mRNA, consequently promoting CRC proliferation and invasion through its interaction with the long intergenic noncoding RNA 460 [32]. Conversely, METTL14, also acting as a “writer”, exerts suppressive effects on tumor cell proliferation and metastasis in vitro and in vivo through M6A modification of the noncoding RNA XIST, a known cancer gene suppressor [33]. 

Regarding CRC metastasis, EMT plays a critical role. Upregulation of METTL3 contributes to EMT by enhancing the expression of miR-1246 and circRNA-1662, thereby promoting CRC metastasis [34, 35]. In contrast, METTL14 negatively regulates the expression of SOX4 through its interaction with the reader protein YTHDF2, leading to its degradation and suppressing the EMT process, invasion, and metastasis in CRC [36]. 

### Demethylases: the erasers of M6A

In addition to writers, M6A modification is regulated by demethylases, referred to as “erasers,” including FTO and ALKBH5. FTO, identified as the first M6A eraser, catalyzes the demethylation of M6A modification. Similarly, ALKBH5 also possesses the ability to remove M6A modification from RNA. The presence of these erasers renders M6A modification a dynamic and reversible process capable of regulating RNA expression and function. Importantly, a study conducted by Ben Yue et al. has revealed that the loss of the M6A demethylase FTO promotes EMT, thereby enhancing tumor metastasis [37]. The findings highlight the significant role of demethylases in the regulation of tumorigenesis and metastasis processes.

### M6A-binding proteins: the readers of M6A

Apart from the “writers” and “erasers,” the proteins known as “readers” of M6A modification, including the YTH domain-containing protein family, HNRNP family, and KH domain family, play crucial roles. These reader proteins possess the ability to recognize and bind to M6A-modified RNA, actively participating in the regulation of RNA translation and degradation processes.

For example, Hao Liu et al. demonstrated that M6A “reader” hnRNPA2B1 plays a significant role in the primary miRNA processing and innate immune response against DNA viruses, exerting essential functions in EMT, drug resistance, and metastasis in CRC and various other cancers [38, 39]. Moreover, M6A modification exerts influence on other key molecules, such as YAP, which significantly promotes the proliferation, invasion, and metastasis of CRC both in vitro and in vivo. Notably, the knockdown of the M6A reader YTHDF3 inhibits YAP-mediated CRC development [40]. These findings underscore the significant regulatory role of M6A modification in the tumorigenesis and metastasis of CRC.

These limited studies indicate that all three factors involved in the M6A modification process contribute to EMT. However, the specific interactions among these factors are still not well understood, and further research is needed to unravel the intricate details of M6A modification and its regulatory factors in EMT. Obtaining more comprehensive information in this regard will help identify potential targets for therapeutic interventions.

## PTM regulation of EMT in CRC

PTM is one of the epigenetic mechanisms that regulate chromatin structure and gene expression, and it plays a significant role in EMT and CRC. Histone modifications typically occur on the four core histones, H2A, H2B, H3, and H4, which form the core structure of nucleosomes and tightly package DNA. PTM can occur at various sites on the N-terminal tails of histones, including methylation, acetylation, ubiquitination, phosphorylation, glycosylation, and amino acid modification. An imbalance in histone modifications has been implicated in tumor development and metastasis [41].

### PTMs involved in the regulation of EMT

#### Methylation

Histone methylation and demethylation are commonly catalyzed by HMTs and HDMs, respectively. Based on the amino acids targeted by the methyltransferases, HMTs can be classified into two families: PKMTs and PRMTs. The PKMT family includes enzymes with SET domains, such as EZH2 and DOT1L, and numerous studies have demonstrated that EZH2 overexpression promotes the proliferation and metastasis of cancer cells, including CRC. Members of the PRMT family also participate in tumor metastasis. For example, PRMT1 has been reported to facilitate the growth and metastasis of melanoma. PRMT1, PRMT5, PRMT6, and PRMT7 have been shown to promote EMT in head and neck cancer, CRC, breast cancer, ovarian cancer, lung adenocarcinoma, and oral cancer cells [42].

#### Acetylation

Protein acetylation refers to the process of adding an acetyl group to lysine residues of proteins, catalyzed by HATs. Conversely, protein deacetylation is mediated by HDACs. Research indicates that SIRT7, as a member of HDACs, serves as a key regulator in TGF-β signaling and can act as an inhibitor for breast cancer metastasis [43]. In contrast, its family member SIRT1, can promote the expression of ZEB1, thereby inducing EMT [44].

#### Ubiquitination

Ubiquitination involves the attachment of ubiquitin molecules to target proteins through a cascade of enzymatic reactions involving ubiquitin-activating enzymes, ubiquitin-conjugating enzymes, ubiquitin ligases, and deubiquitinases. This modification facilitates the selective degradation or regulation of target proteins in the cell. USP10, as a deubiquitinase, inhibits ZEB1-mediated EMT and CRC metastasis by regulating ZEB1 ubiquitination and promoting its proteasomal degradation [45].

#### Phosphorylation

Phosphorylation and dephosphorylation, catalyzed by kinases and phosphatases, respectively, involve the addition or removal of phosphate groups to proteins or other molecules. Phosphorylation is one of the most common chemical modifications in the human proteome, with over 90% of encoded proteins being phosphorylated. A study suggests that phosphorylation can control the expression of SNAIL [46]. Additionally, phosphorylation of CAP1 has been shown to promote EMT [47].

#### SUMOylation

It has been shown that SUMOylation plays an important role in regulating transcriptional activity, inhibiting the function of TFs, and influencing the transition between epithelial and mesenchymal differentiation states. Therefore, drugs targeting SUMOylation have been considered to have the potential to alter cancer differentiation states [48].

#### Glycosylation

Glycosylation enhances the stability and function of EZH2 protein through the miR-101-O-GlcNAc/EZH2 feedback loop, promoting EMT and driving CRC metastasis. Therefore, the inhibition of O-GlcNAcylation also has been proposed as a potential strategy for treating metastatic CRC [49].

### Key pathway for the PTM regulation of EMT

As a core factor in EMT, TGF-β plays a key role in the occurrence and metastasis of CRC by inducing immune escape, promoting angiogenesis, and driving the EMT. Notably, the SMAD complex, represented by SMAD4, acts as a crucial partner in the activation of the TGF-β signaling pathway. The function of SMAD proteins is extensively regulated and controlled by various PTMs, including methylation, phosphorylation, ubiquitination, and SUMOylation. For instance, phosphorylation of SMADs is a critical event triggered by TGF-β signaling, and PRMT5 can methylate SMAD4 at the R361 site, increasing TGF-β pathway activity and promoting EMT and CRC metastasis [6, 50]. O-GalNAcylation has been demonstrated to regulate TGF-β to suppress the EMT process, thereby inhibiting cancer cell migration and invasion [51]. Another study demonstrated that EZH2-mediated methylation of SMAD3 at K53/K333 can drive cancer metastasis [52]. Furthermore, traditional understanding suggests that SMAD signaling is negatively regulated by ubiquitination-mediated degradation, which reduces the activity of the TGF-β pathway and inhibits EMT [53]. However, in the study conducted by Jianyin Long et al., they found that both mono-ubiquitination and mono-SUMOylation of Smad4 can enhance protein stability and TGF-β signal transduction, while global ubiquitination may inhibit nuclear Smad signaling in a gene-specific manner. Depending on the target promoter being examined, the impact of Smad4 SUMOylation can exhibit a diverse outcome, highlighting the complexity of Smad ubiquitination’s impact on TGF-β superfamily signaling [54]. Numerous studies have reported that PTMs can regulate the process of EMT by modulating the activity of key EMT-TFs [43, 55]. TGF-β has long been considered a promising target for EMT therapy, and SMAD proteins can indirectly influence EMT by regulating the activity of the TGF-β signaling pathway through various PTMs. Furthermore, another study revealed that PBX4 as an effector of this pathway, overexpression upregulates important EMT molecules and significantly increases the expression of the angiogenesis marker VEGF-A, suggesting a potential role of PBX4 in promoting CRC angiogenesis. The overexpression of PBX4 is likely influenced by PTMs and further research is needed to confirm its therapeutic potential [56] (Fig. 2).

## Therapeutic applications of epigenetic regulation of EMT in CRC

Combining epigenetic modifiers with conventional chemotherapy or radiotherapy has shown promising results in enhancing treatment sensitivity. Consequently, it has emerged as a focal point in cancer research, providing new avenues for breakthroughs and therapeutic strategies [57]. A multitude of epigenetic modifiers, such as DNMT inhibitors, HDAC inhibitors, and EZH2 inhibitors, have been developed for cancer prevention, treatment, and overcoming drug resistance. For instance, selective METTL3 inhibitors have recently exhibited remarkable therapeutic efficacy in acute myelogenous leukemia [58]. However, it should be emphasized that CRC epigenetic therapy has demonstrated limited clinical response when used as a standalone approach and is notably less effective than when combined with other drugs [59]. Further investigation is therefore warranted to elucidate the optimal dosage and combination regimens for optimal outcomes.

Here, we discuss the involvement of EMT in mediating drug resistance and immune therapy mechanisms in CRC. Additionally, we explore the relevant inhibitors targeting EMT in these contexts.

### Drug resistance in CRC

#### M6A modification

Given that M6A modification is primarily catalyzed by the METTL3-METTL14 methyltransferase complex, targeting methyltransferase presents an evident approach for developing drugs related to M6A modification. Furthermore, CSCs, known for their self-renewal and differentiation capacities, contribute to tumor recurrence and exhibit resistance to chemotherapy and radiation. Investigations have linked CSC-associated chemotherapeutic resistance to the activation of the Wnt/β-catenin pathway mediated by METTL3 [60, 61]. Additionally, interference with METTL3 has been shown to disrupt the interaction between TCF7L2 mRNA and hnRNPA2B1, resulting in reduced TCF7L2 expression and preventing TCF7L2 from enhancing resistance to antibody therapy [50, 62]. Consequently, selective METTL3 inhibitors hold potential as a therapeutic strategy to overcome drug resistance in CRC.

#### PTM modification

PTM plays a significant role in the drug resistance of CRC. 5-FU is a frequently used chemotherapy agent for advanced or high-risk recurrent CRC, but its effectiveness in CRC cells is influenced by multiple factors. For instance, a recent study demonstrated that the glycosylated cell surface transmembrane protein CD147 of the IgSF promotes EMT and tumor metastasis by activating the MAPK/ERK pathway, consequently reducing CRC cell sensitivity to 5-FU [63]. However, another intriguing study indicates that 5-FU treatment activates the TGF-β pathway, which provides protective effects against 5-FU toxicity by regulating the microenvironment and cellular mechanisms related to cell death and proliferation genes [64]. This establishes TGF-β as a crucial regulator of 5-FU resistance. TGF-β, being a key player in EMT, is subject to a wide range of direct and indirect posttranslational modifications, making the regulation of TGF-β signaling through posttranslational modifications a promising strategy to restore chemosensitivity [64]. Similar pathways, such as NF-κB, Wnt, FGF, and EGF/HER2, have been found to be activated during chemotherapy or radiation therapy, stimulating the expression of EMT-TFs [65]. As mentioned earlier, EMT-TFs and related signaling pathways are widely regulated by direct or indirect PTMs. Therefore, investigating the mechanisms of PTMs holds the potential to restore cellular sensitivity to chemotherapy and enhance chemosensitivity.

Although epigenetic drugs can serve as sensitizers, translating these preclinical findings into clinical tumor treatment remains challenging due to the pleiotropic nature of their effects on normal cells. For instance, glycosylation inhibitors are widely utilized in chemotherapy. However, abnormal glycosylation is also implicated in various mechanisms of anti-cancer treatment resistance. These mechanisms include alterations in drug absorption, disruption of signal activation, resistance to apoptosis, acquisition of CSC properties, and changes in drug metabolism, which ultimately limit the efficacy of glycosylation inhibitors [66]. Further research is necessary to address the pleiotropic effects of epigenetic modifiers, which pose significant barriers to their application in clinical settings.

### Immunotherapy of CRC

Tumor immunotherapy is based on the study of tumor escape mechanisms and aims to activate human anti-tumor immune responses, thereby killing tumor cells and overcoming tumor evasion pathways [67]. Epigenetic modulators offer a promising approach to enhance the response to immune checkpoint blockade through various mechanisms, such as promoting chemokine expression on T cells and reducing the presence of suppressor cells in the TME. Manipulating the host immune response using epigenetic drugs holds significant potential for cancer immunotherapy. For instance, EZH2, a histone methyltransferase, plays a pivotal role in tumor development and metastasis [68]. Selective inhibitors of EZH2 methyltransferase activity, like ESK126, have demonstrated the ability to synergistically enhance the therapeutic efficacy of T cell therapy while increasing the infiltration of CXCL9 and CXCL10 chemokines, as well as CD8^+^ T cells [69]. Furthermore, the combination of histone methyltransferase inhibitors with other epigenetic drugs, such as simultaneous inhibition of DNMT and LSD1, has shown synergistic effects in reactivating epigenetically silenced genes in cancer cells [70]. These studies provide encouraging evidence for the potential of combining epigenetic therapy with immunotherapy in the treatment of CRC.

The EMT process is tightly regulated by PTMs and M6A modifications. Notably, on the one hand, EMT is closely associated with the generation of CSCs [71]. On the other hand, its occurrence predominantly takes place in the invasive front of metastatic tumors, making it highly susceptible to factors from the microenvironment, including immune factors, chemokines, cytokines, and growth factors. The study by Richard C. Bates et al. has demonstrated that TGF-β, VEGF, and TNF-α promote malignancy in CRC cells by inducing EMT. These insights can be utilized to explore potential immunotherapy strategies focusing on EMT for CRC [26].

#### M6A modification

Mettl3-mediated M6A modification not only promotes the activation of DCs and M1 macrophages but also facilitates the expression of PSMC5, inducing EMT to prompt cancer cell metastasis and increasing infiltration of M2 macrophages and N2 neutrophils. For instance, investigations have revealed that M2 macrophages are capable of facilitating the process of EMT through the upregulation of CRYAB expression and activation of the ERK1/2/Fra-1/slug signaling pathway, thereby promoting invasion and metastasis of CRC [72]. Notably, this process may result in metastasis to distant sites, such as the lung. Furthermore, a scientific study revealed that inhibiting M6A modification through the consumption of the methyltransferase enzymes METTL3/14 can potentiate the response of CRC subtypes to PD-1 immunotherapy, augmenting the efficacy of immune checkpoint blockade in treating CRC. These evidences emphasize the potential of METTL3 inhibitors as a therapeutic approach for CRC treatment again [73]. Concomitantly, the knockout of the “eraser” FTO gene has been demonstrated to diminish the stability of STAT1 and PPAR-γ, consequently impeding the polarization of M1 and M2 macrophages [74]. Research has shown that FTO can directly upregulate LILRB4 via the M6A mechanism, promoting tumor invasion and suppressing T-cell activity. In light of these observations, therapeutic interventions targeting the FTO/M6A axis have exhibited a notable capacity to impede the self-renewal and immune evasion mechanisms of CSCs [75, 76]. Small molecule inhibitors of FTO have emerged as promising strategies for cancer Immunotherapy.

#### PTM modification

In turn, immune factors have the capability to induce PTMs that exert bidirectional regulatory effects. For instance, IL-6 stimulation has been shown to initiate the EMT process in CRC cells while concurrently inducing acetylation at K685 and phosphorylation at Y705 of STAT3. Remarkably, elevated acetylation levels of STAT3 within tumor tissues can bind to DNMT1, resulting in the inhibition of tumor suppressor genes via promoter CpG island methylation [77]. This molecular alteration further intensifies the aggressiveness of CRC.

### Small molecule inhibitors of CRC

Recently, there has been a significant increase in the development and research of epigenetic modifiers, particularly inhibitors, as potential therapeutic drugs. The growing understanding of the involvement of epigenetic modifications in EMT has provided a theoretical basis for the development of related inhibitors. Some modifiers are still in the theoretical stage, while many others are currently undergoing or are about to enter preclinical trials, bringing new advancements in tumor treatment for CRC.

#### Inhibitors of chromatin readers that recognize histone acetylation

Thiery et al. have designed small molecule compounds specifically as inhibitors of genes that promote EMT. These inhibitors target specific binding proteins that recognize various histone modifications, including the acetylation-histone binding BD. By disrupting the interaction between BD-containing transcriptional co-activators and acetylated lysine residues, these inhibitors can modulate the expression of key EMT genes and lead to a reduction in the metastatic potential of CRC in mouse models. Specifically, the expression of the epithelial marker E-cadherin was increased, while the expression of the mesenchymal marker vermentin was decreased [78–80].

#### FXR inhibitors

FXR is a nuclear receptor activated by bile acids and involved in bile acid metabolism. A recent systematic review of FXR studies found that FXR activation not only significantly affects immune cell function and the release of chemokines but also inhibits EMT [81]. FXR is subject to a wide range of PTMs, such as acetylation, ubiquitination, and methylation. This indicates that FXR has the potential to act as a tumor inhibitor by regulating tumor cell proliferation and invasion through epigenetic regulation.

#### DOT1L and CBP inhibitors

SNAIL and ZEB1, as important TFs of EMT, along with DOT1L/KMT4, a non-SET domain methyltransferase, have been studied extensively. Research has revealed that high expression of DOT1L/KMT4 leads to elevated methylation levels of H3K79 around the SNAIL and ZEB1 promoters, resulting in the repression of CDH1 transcription, which is associated with EMT [82]. Consequently, inhibitors targeting DOT1L have been designed to impede H3K79 methylation, effectively inhibiting EMT progression [83]. The acetylation of DOT1L mediated by CBP plays a pivotal role in maintaining the stability of DOT1L. This stability ensures proper H3K79 methylation, expression of EMT-TFs, and subsequent migration and invasion in CRC [84]. These findings underscore the potential therapeutic targets of CBP inhibitors and DOT1L inhibitors when used in combination.

#### EZH2 inhibitors

EZH2, functioning as a histone methyltransferase, plays a crucial role in epigenetic regulation and the induction of EMT, making it a potential target for epigenetic therapy [85]. Clinical trials are underway for multiple EZH2 methyltransferase inhibitors, and recent studies have shown that AMPK agonists hold promise as sensitizers for EZH2-targeted cancer therapy, enhancing the efficacy of EZH2 inhibitors [86]. Furthermore, the combination of EZH2 methyltransferase inhibitors with ERK inhibitors effectively inhibits TGF-β-induced EMT, further emphasizing the potential of EZH2 methyltransferase inhibitors.

#### DNMT inhibitors

DNMTs, including DNMT1, DNMT3A, and DNMT3B, serve as the core enzymes for DNA methylation and are responsible for guiding the methylation patterns of the mammalian genome. Due to the important role of DNA methylation in tumorigenesis and metastasis, it has received widespread attention and research. Xianmei et al. pointed out that DNMT3B plays a critical role in the progression from normal epithelium to dysplastic epithelium. DNMT inhibitors have the potential to interrupt the progression from adenoma to carcinoma and inhibit EMT as epigenetic regulators [87]. However, it is worth noting that research has indicated that treatment of MCF-7 breast cancer cells with the DNMT inhibitor 5-aza-2’-deoxycytidine to maintain a low methylation state resulted in the upregulation of the expression levels of EMT-associated genes involved in invasion and enhanced the invasive and migratory capabilities of the cells. This suggests that DNMT inhibitors may have complex regulatory mechanisms on EMT and could potentially increase the risk of tumor cell migration and dissemination [88].

## Discussion

In recent years, research focusing on various epigenetic modifications and their respective target points has provided a rich theoretical foundation for understanding the pathogenesis and therapeutic strategies of colorectal cancer. Through our discussions, it has been revealed that EMT is not only closely associated with tumor metastasis but also interacts with the tumor microenvironment, inducing the generation of tumor stem cells and circulating tumor cells. This article emphasizes that M6A and PTM modifications of EMT in colorectal cancer will play a crucial role in the occurrence, metastasis, and treatment of tumors. Specifically, EMT-related transcription factors and pathways, as targets, are extensively regulated by epigenetics such as the TGF-β pathway and ZEB family. These intricate regulatory mechanisms may provide new directions for understanding tumor resistance and treatment.

On one hand, epigenetic modifiers such as DNMT inhibitors, HDAC inhibitors, EZH2 inhibitors and FXR inhibitors have shown potential effects in disrupting the EMT process, influencing the tumor microenvironment, especially regulating immune cell function and chemokine release, and inhibiting tumor progression. On the other hand, under PTM modification with 5-FU and M6A regu1lation with CSCs, the modulation of drug resistance through influencing the EMT process has been demonstrated, revealing potential therapeutic strategies for restoring chemotherapy sensitivity and overcoming colorectal cancer resistance.

Overall, epigenetic regulation of the EMT process plays a crucial role in colorectal cancer resistance mechanisms, drug treatment, and immunotherapy. This impact is not limited to colorectal cancer but also holds significant potential in other tumor domains. However, due to safety considerations, the clinical application of epigenetic modifiers remains limited, and its true potential awaits systematic exploration and validation. Additionally, further research and discussion are needed to understand the impact of other important epigenetic modifications on EMT, including DNA methylation and non-coding RNAs.

## Data Availability

The authors confirm that the data supporting the findings of this study are available within the article.

## Abbreviations

5-FU: 5-Fluorouracil
ALKBH5: AlkB homolog 5
AMPK: AMP-activated protein kinase
APC: Adenomatous polyposis coli
BD: Bromine domain
BMI1: B-lymphoma Mo-MLV insertion region 1 homolog
BRAF: B-Raf proto-oncogene, serine/threonine kinase
CAP: Cyclase-associated protein
CDH1: Cadherin 1
CRC: Colorectal cancer
CRYAB: Crystallin alpha B
CSC: Cancer stem cell
CTC: Circulating tumor cell
CXCL: Chemokine (C-X-C motif) ligand
DC: Dendritic cell
DNMT: DNA methyltransferase
DOT1L/KMT4: Disruptor of telomeric silencing 1-like histone lysine methyltransferase
ECM: Extracellular matrix
EGF/HER2: Epidermal growth factor/ human epidermal growth factor receptor 2
EMT: Epithelial-mesenchymal transition
EpCAM: Epithelial cell adhesion molecule
ERK: Extracellular signal-regulated kinase
EZH2: Enhancer of zeste homolog 2
FAS: Fas cell surface death receptor
FGF: Fibroblast growth factor
FTO: Fat mass and obesity-associated protein
FXR: Farnesoid-X receptor
HAT: Histone acetyltransferase
HDAC: Histone deacetylase
HDM: Histone demethylase
HMGA1: High-mobility group AT-hook 1
HMT: Histone methyltransferase
HNRNP: Heterogeneous nuclear ribonucleoprotein
IgSF: Immunoglobulin superfamily
IL: Interleukin
KH: K homology
KRAS: Kirsten rat sarcoma viral oncogene homolog
LGR: Leucine-rich repeat-containing G protein-coupled receptor
LILRB4: Leukocyte Immunoglobulin-like Receptor B4
LSD1: Lysine-specific histone demethylase 1
M6A: N6-methyladenosine
MAPK/ERK: Mitogen-activated protein kinase/extracellular signal-regulated kinase
METTL: Methyltransferase-like

## Code availability

The authors confirm that the code of this study is available within the article.
